
JOURNAL INFORMATION
==============================
NLM Title Abbreviation: ScientificWorldJournal
Journal ID: ScientificWorldJournal
NLM Journal ID: 101131163
Journal ID: TSWJ

The Scientific World Journal

ISSN: 2356-6140
EISSN: 1537-744X
Publisher: Wiley

ARTICLE INFORMATION
==============================
PMCID: PMC6009525
PMID: 29973816
DOI: 10.1100/tsw.2002.42
Article ID: 526914
Article version: 1
Subjects: Short Report

Electron Tomography: Towards Visualizing Supramolecular Architecture Inside Cells

Baumeister W. *
Medalia O.
Boehm J.
Nickell S. St.
Frangakis S. A.
Max-Planck-Institute of Biochemistry, Am Klopferspitz 18a, D-85152 Martinsried, Germany
*W. Baumeister: baumeist@biochem.mpg.de
Publication date: 2002
Electronic publication date: 2002 Jan 5
Volume: 2
First page: 89
Last page: 90
Copyright: Copyright © 2002 W. Baumeister et al.
Copyright year: 2002
License: This is an open access article distributed under the Creative Commons Attribution License, which permits unrestricted use, distribution, and reproduction in any medium, provided the original work is properly cited.
License URL: https://creativecommons.org/licenses/by/3.0/

==============================
Advances in Gene Technology: The Genome and Beyond –
Structural Biology for Medicine (Proceedings of the 2002 Miami
*Nature Biotechnology* Winter Symposium)
*TheScientificWorld* 2002, 2(S2), 89–90
ISSN 1532-2246; DOI 10.1100/tsw.2002.42

### ELECTRON TOMOGRAPHY: TOWARDS VISUALIZING SUPRAMOLECULAR ARCHITECTURE INSIDE CELLS

W. Baumeister*, O. Medalia, J. Boehm, St. Nickell, and A. Frangakis
Max-Planck-Institute of Biochemistry, Am Klopferspitz 18a, D-85152 Martinsried, Germany
*e-mail: baumeist@biochem.mpg.de

**INTRODUCTION.** It has long been a dream of cell biologists to catch a glimpse of the molecular architecture inside living cells, so far largely an uncharted territory. In addition to relatively robust molecular machines, these are undoubtedly many ensembles of macromolecules which jointly perform vital functions, but are held together by forces too weak to withstand conventional biochemical isolation and purification procedures[1,2].

**METHOD.** Electron tomography is the most general method for obtaining 3-D information by EM. It can be applied to pleomorphous structures like organelles, whole cells or tissue sections embedded in vitreous ice, i.e. without chemical fixation or staining.

The basic concept is not new, but formidable technical obstacles prevented realization of its full potential until recently. The key problem is to reconcile two conflicting requirements: In order to obtain a detailed and undistorted reconstruction, a tilt series must be recorded that covers as wide a tilt range as possible in as many increments as possible. At the same time, the electron dose must be kept subcritical if radiation damage is not to erase structural details[3].

The development of automated electron tomography has made it possible to obtain 3-D reconstructions of whole ice-imbedded prokaryotic cells or organelles such as mitochondria with resolutions in the range of 6 to 8 nm and with advanced instrumentation we are now entering the realm of molecular resolution (2 to 4 nm). In spite of the inevitably low signal-to-noise ratio of the tomograms, it is possible to detect and identify macromolecular complexes by virtue of their structural signature, provided that high-to-medium resolution structures of the molecules under scrutiny are available to be used as templates in searching the reconstructed volumes. The search can, of course, be performed with multiple templates, allowing not only the mapping of territorial distributions, but also revealing spatial relationships in functional "neighborhoods", thus bridging the gap between molecular and cellular structural biology[4].

**RESULTS.** In order to demonstrate the feasibility of the approach and to assess the level of fidelity in mapping specific macromolecules we have performed extensive studies with 'phantom cells'; there are liposomes of known macromolecular contents. Studies with real cells include several archaea and dictyostelium cells grown directly on EM grids. Beyond visualizing the cytoskeleton with a noninvasive method and with unprecedented resolution, it has also been possible to detect and identify several macromolecular complexes *in situ*.

### REFERENCES

1. Baumeister, W., Grimm, R., and Walz, J. (1999) Trends Cell Biol. 9, 81–85.
2. Baumeister, W. and Steven, A.C. (2000) Trends Biochem. Sci. 25, 624–631.
3. Koster, A., Grimm, R., Typke, D., Hegerl, R., Stoschek, A., Walz, J., and Baumeister, W. (1997) J. Struct. Biol. 120, 276–308.
4. Boehm, J., Frangakis, A.S., Hegerl, R., Nickell, S., Typke, D., and Baumeister, W. (2000) Proc. Natl. Acad. Sci. U. S. A. 97, 14245–14250.
